# Cardiovascular Risk After Concussion: An Overlooked Dimension in Athletes’ Risk Stratification

**DOI:** 10.1016/j.jacadv.2025.101949

**Published:** 2025-07-03

**Authors:** Ioannis Skalidis, Panagiotis Antiochos

**Affiliations:** a Interventional Cardiology Department, Institut Cardiovasculaire Paris-Sud, Hôpital Jacques Cartier, Ramsay-Santé, Massy, France; b Lausanne University Hospital, Lausanne, Switzerland

The recent study by Rim et al^1^ provides important prospective evidence linking sport-related concussions with cardiovascular risk among collegiate American-style football athletes. The authors demonstrate that concussions are independently associated with arterial stiffening, increases in both systolic and diastolic blood pressure, and the emergence of maladaptive cardiac phenotypes such as increased left ventricular mass and reduced diastolic function. These findings underscore the potential for concussion to act not only as a neurologic event but also as a novel cardiovascular risk factor.

The strength of this study lies in its longitudinal design, careful control matching, and multimodal cardiovascular assessments, including echocardiography and pulse wave velocity measurements. By extending beyond neurocognitive endpoints, the authors challenge us to reconsider how concussions are understood within broader systemic frameworks. Could the pathophysiologic sequelae of concussion—via autonomic dysregulation or persistent neuroinflammation—initiate or accelerate vascular aging and remodeling?

The implications of these findings are particularly salient given the high prevalence of hypertension and cardiovascular dysfunction already documented in American-style football athletes. Importantly, the observed changes were not fully explained by known contributors such as weight gain or player position, strengthening the case for concussion as an independent modifier of cardiovascular health.

This study also raises key clinical questions: should cardiovascular monitoring be integrated into postconcussion care algorithms? And if so, what duration and modality of follow-up would be appropriate to detect and mitigate evolving risk? Incorporating blood pressure surveillance into return-to-play protocols may represent a low-cost, high-yield strategy—especially considering the treatable nature of hypertension and its known links to later-life cognitive decline.

Rim et al^1^ provide compelling data that broaden the lens through which we view concussion. Future research should investigate whether early cardiovascular intervention postconcussion can reduce long-term morbidity, potentially transforming how we manage both the athlete's heart and brain.
